# Reported prevalence and risk factors of malignant tumors in the elderly population in China: a nationwide cross-sectional study

**DOI:** 10.1007/s40520-025-03109-1

**Published:** 2025-06-23

**Authors:** Tianjie Li, Dan Jiang, Meng Fu, Xinye Qian, Zhong Wang, Haifeng Song, Deping Liu, Jianxing Li, Xiaodong Liu

**Affiliations:** 1 Department of Urology, The First Affiliated Hospital of Kunming Medical University, No. 295, Xichang Road, Kunming City, Yunnan Province China; 2 Department of Urology, Beijing Tsinghua Changung Hospital, 168 Litang Rd, Changping District, Beijing, Zip Code:100730 China; 3 Department of General Medicine, Beijing Tsinghua Changung Hospital, Beijing, China; 4 Graduate School of Peking Union Medical College, Chinese Academy of Medical Sciences, No.9, Dongdan Santiao, Dongcheng District, Beijing, 100730 China

**Keywords:** Prevalence, Risk factors, Malignant tumors

## Abstract

**Background:**

Malignant tumors are one of the most challenging public health problems facing mankind. It is of great significance to systematically, comprehensively, and fully understand and analyze the prevalence and distribution of malignant tumors in the elderly population.

**Aims:**

To evaluate the prevalence and associated factors of malignant tumors in the elderly population in China, and quantify the differences in prevalence and associated factors by urban and rural area, sex, and age.

**Method:**

The data is based on Chinese citizens aged 60 and above, using a self-weighted sampling design of stratified, multi-stage, Probability Proportionate to Size Sampling (PPS), and equal-probability sampling at the end of the stage. The analysis included 224,142 valid questionnaires.

**Results:**

Among the 215,041 elderly people who participated in the survey, a total of 2,463 participants reported the diagnosis of malignant neoplasms, with a prevalence rate of 1.2%. Urban and rural areas, marital status, living alone, drinking, medical insurance, income, education level and economic status were associated with malignant tumors. Rural household registration, living alone, moderate alcohol consumption, without medical insurance and having income were associated with a lower prevalence of malignant tumors.

**Conclusions:**

The prevalence of malignant tumors in urban residents is higher than that in rural residents. There was no significant difference in the overall prevalence of malignancies between sexes. Urban residence, not living alone, higher education level, and poor economic conditions were found to be associated with a higher prevalence of malignant tumors among the elderly, while widowhood and moderate alcohol consumption appeared to be associated with lower prevalence.They could inform future prevention strategies for malignancies and highlight unresolved health disparities.

**Supplementary Information:**

The online version contains supplementary material available at 10.1007/s40520-025-03109-1.

## Introduction

Malignant tumors constitute a significant global threat to human life, leading to substantial socio-economic repercussions and representing one of the foremost public health challenges of our era [1]. Noticeable discrepancies exist in the prevalence and mortality rates of cancer, influenced by both sex and geographical location [2]. China is experiencing an escalating trend in the mortality and morbidity rates of malignant tumors, which have now become the leading cause of death among its population [3]. There are variations in the prevalence, mortality, and treatment status of malignant tumors among different countries and regions worldwide, with multiple factors contributing to these differences, including ecology, environment, population, culture, age distribution, economic level, and genetic factors, among others [4]. Common risk factors for malignant tumors include smoking, alcohol consumption, lack of physical activity, obesity, malnutrition, and chronic infections, among others [5]. Furthermore, studies have shown that age is the most significant independent risk factor for the occurrence and mortality of malignant tumors. This is because, to a certain extent, as individuals live longer, they are exposed to carcinogens and genetic damage for a longer duration. Additionally, cellular aging brings about a series of physiological changes that increase the likelihood of developing malignant tumors [6]. Furthermore, with age comes an increase in concurrent health issues and physical deterioration, which curtail an individual’s tolerance to treatment, resulting in poorer treatment efficacy, protracted treatment durations, elevated costs, and less favorable prognoses [7]. Demographic factors such as sexual orientation and sex significantly influence cancer prevalence rates. Research shows that sexual minority youths, including homosexual, bisexual, and transgender individuals, exhibit a higher prevalence of cancer-related risk behaviors compared to their heterosexual peers. These behaviors, such as substance use, sexual activity, and dietary patterns, are linked to higher cancer prevalence, suggesting a greater lifetime cancer risk for sexual minorities [8]. Specifically, sexual minority women could have an increased breast cancer risk compared to heterosexual women [9]. Genetic and epigenetic factors, influenced by sex chromosomes, also contribute to disparities in cancer risk between males and females. Cultural, economic, and lifestyle differences further shape these disparities. Therefore, it is important to consider factors such as sex, sexual orientation, and their associated risk behaviors when developing cancer prevention strategies, as these factors may have significant implications for cancer prevalence.

The average age of cancer onset varies significantly across the globe. In developed countries or regions, it stands at 66.38 years, while in less developed areas, it drops to 61.75 years [10]. In China, the average age is 63.47 years. A United Nations Development Programme (UNDP) report reveals a correlation between the Human Development Index (HDI) and the average age of cancer onset, with higher HDI correlating to higher average age. However, the discrepancies diminish when adjustments are made for population age structure. From a geographical perspective, Africa reports the youngest average age of onset at 51.63 years, while Europe reports the oldest, at 67.03 years. According to 2014 data from the National Cancer Registration Center [11], urban regions in China display a higher incidence of malignant tumors than rural regions. Despite decreases after adjusting for age structure, urban areas still exhibit higher rates. While urban areas have greater mortality rates, these rates drop below rural areas’ rates once age structure is considered. Variations in morbidity might be associated with differences in diet and behavioral habits, while the influence of regional medical resources and economic income may play an underlying role. Statistical evidence reveals the incidence of malignant tumors to be at its nadir within the 35–39 age group and progressively increases with age, culminating in a peak among the 80–84 age group [12]. The distribution of age-specific incidence rates varies depending on the type of malignant tumor. For example, the average age at diagnosis for breast cancer in women is 45–55 years, with the peak incidence estimated at 50–54 years, while liver cancer is diagnosed at a higher average age. Among lung cancer patients, the 65–70 age group has the highest proportion [13]. In terms of digestive system malignant tumors, the risk of gastric cancer [14]and esophageal cancer [15] increases in Chinese adults aged 40 and above, while the incidence of colorectal cancer significantly increases from the age of 50 [16]. Similarly, malignant tumor mortality rates also exhibit age-specific patterns, showing a continuous increase with age [17]. With the aging population, the proportion of elderly cancer patients has significantly increased [18, 19].

However, current research specifically targeting the prevalence and distribution characteristics of malignant tumors in older individuals, especially large-scale, nationwide epidemiological studies, is noticeably limited. This study intends to delve deeply into the prevalence of malignant tumors in elderly patients across various classes, guided by a stratified analysis of specific factors like sex and age. Our aim is to enhance understanding of the epidemiological patterns and socioeconomic characteristics of malignant tumors in the elderly. This research will serve as a scientific and factual foundation for devising comprehensive strategies for the prevention and treatment of malignant tumors and fostering healthy aging. The significance of this endeavor is profound.

## Materials and methods

### Study participants and data source

The data analyzed in this study are from the fourth “Sample Survey of the Aged Population in Urban and Rural China” project (SSAPUR) survey, which is a cross-sectional survey. The survey targeted Chinese citizens aged 60 and above and employed a stratified, multi-stage, probability proportionate to size sampling (PPS) design with self-weighting sampling. The sampling process involved four stages: In the first stage, the sample size was allocated to each province (city, autonomous region) based on the proportion of the elderly population to the national population. A total of 466 districts (counties) were selected from 2,853 districts (counties) across the 31 provinces (cities, autonomous regions) of mainland China. In the second stage, four townships (streets) were selected from each district (county) using PPS sampling based on the total elderly population in each district (county) as auxiliary information. In the third stage, four villages (neighborhood committees) were selected from each township (street) using PPS sampling based on the total elderly population in each township (street) as auxiliary information. In the fourth stage, 30 elderly individuals were selected using equal interval sampling from the list of elderly individuals obtained before the survey in the selected townships (streets). The designed sample size for this survey was 223,680, with a sampling ratio of approximately one in a thousand. The survey employed home visits and questionnaire-based interviews to collect information on nine aspects, including the basic and family conditions, health status, and care needs of the elderly. If a selected elderly individual refused to participate in the survey, was deceased, relocated, unreachable (after at least three contact attempts), or resided in long-term care facilities, they were excluded, and new participants were sequentially selected from the candidate list. The survey was conducted from August 1st to August 31st, 2015, and the final number of valid questionnaires was 224,142. SSAPUR is the largest aging-related database in China to date.

This study has obtained approval from the Ethics Review Committee of Beijing Hospital (No. 2021BJYYEC-294-01) and the National Bureau of Statistics (No. [2014] 87). Written informed consent was obtained from all participants.

### Grouping and variable settings

The data collection was overseen by skilled researchers adhering to a standardized protocol. Trained professionals conducted face-to-face interviews with elderly community residents to complete all questionnaires, aiming to mitigate recall bias. These experts diligently elucidated each questionnaire item to ensure the reliability and accuracy of the respondents’ responses. Each questionnaire had a unique identification number, start and end time, and the signature of the investigator on the cover. Due to the large amount of collected information, unnecessary details were removed, and the following population characteristics, health, social participation, family life, and psychological information were retained.

The demographic characteristics include sex, age, education level, urban/rural status, and marital status. The age category was determined based on the individual’s identification card. If the elderly person did not have an ID card, it was filled out by the elderly person or their family member during the interview and divided into three groups: 60–69 years, 70–79 years, and > 80 years. Urban/rural information was determined based on the agricultural or non-agricultural household registration recorded in the household register or determined by the interviewers. Medical security refers to the national healthcare insurance system (including Urban Employee Basic Medical Insurance, Urban Resident Basic Medical Insurance, and the New Rural Cooperative Medical Scheme) as well as other forms of health insurance, such as commercial insurance, categorized into ‘’yes’’ and ‘’no’’. Public welfare activities include maintaining community public order, assisting in resolving neighborhood disputes, preserving the community’s sanitation environment, helping neighbors, caring for the education of the younger generation (excluding the education of one’s own grandchildren), and participating in cultural and scientific promotion activities. Participation is classified as ‘’participated’’ if the individual has participated at least once, and ‘’nonparticipated’’ if no participation occurred. Economic conditions are based on the self-assessment of elderly respondents, classified into five categories: ‘’ very rich’’ ‘’ better rich’’ ‘’ basically enough’’ ‘’ poorer’’ and ‘’ very poor’’. External abuse refers to physical, emotional, or sexual abuse originating from outside the household (e.g., from schools, communities, or strangers), categorized as ‘’yes’’ and ‘’no’’. Spiritual and cultural life encompasses activities such as watching TV/listening to the radio, reading books/newspapers, attending cinemas or theaters, practicing Tai Chi/fitness, playing gateball/table tennis/badminton, or engaging in Mahjong/cards/chess, divided into ‘’yes’’ and ‘’no’’. Malignant tumors were self-reported by respondents as tumors diagnosed by healthcare institutions.

### Statistical analysis

The prevalence rate of malignant tumors will be described. Baseline characteristics and other factors will be presented as count data. When comparing two or more groups, the chi-square test for categorical variables and post-hoc two-tailed Newman-Keuls test will be used to assess the statistical significance of differences. In the univariate logistic regression analysis, demographic data (age, sex, urban/rural status, education level), lifestyle habits (smoking, exercise), and socio-economic factors (medical insurance, paid employment, economic status, public welfare activities, mental and cultural activities) will be analyzed as independent variables. A p-value of < 0.05 will be considered statistically significant. Variables that are statistically significant in the univariate logistic regression analysis will be included in the multivariate logistic regression analysis to identify factors associated with the prevalence of malignant tumors in the elderly. Stratified analysis will be conducted based on sex, age, and urban or rural residence. The chi-square test will be used to analyze the prevalence of malignant tumors after stratification. A p-value of < 0.05 will be considered statistically significant. The lower and upper limits of the 95% confidence interval for the proportion of malignant tumors will be calculated using Robert Newcombe’s method. All statistical analyses will be performed using SPSS 24.0 (IBM Corporation, Armonk, NY, USA).

## Results

### Prevalence of malignant tumors in elderly population by provinces in China

**Fig. 1:**
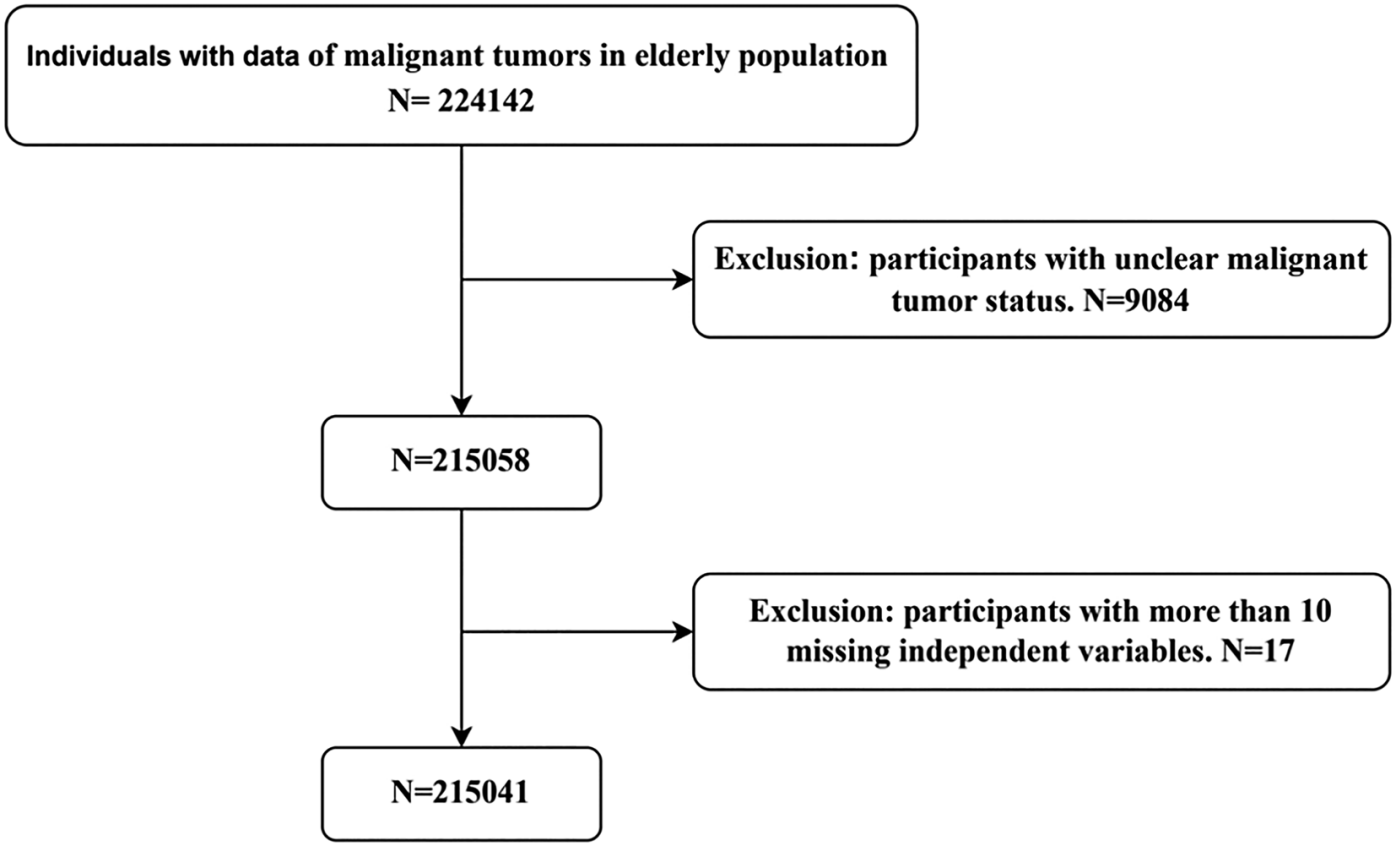
The flowchart of this study

Out of a total of 224,142 collected data, we excluded 9,084 participants with unclear malignant tumor status and 17 participants with more than 10 missing independent variables. Finally, data from 215,041 participants were included in this study. Among them, there were 102,692 male participants and 112,349 female participants.(Fig. 1).

At the time of the survey (August 2015), 2,463 cases of malignant tumors were reported among 215,041 elderly participants, resulting in a point prevalence of 1.2%. The number of participants and the prevalence of malignant tumors in each of the 31 provinces in China are listed in Table 1.

**Table 1:** Number of participants and prevalence of malignant tumors in 31 provinces in China

Province	Number of people without malignant tumors	Number of people with malignant tumors	prevalence rate
Shanghai	4168	128	3.0%
Beijing	3266	93	2.8%
Xinjiang Uygur Autonomous Region	2336	42	1.8%
Jiangsu	15,355	274	1.8%
Hubei	3502	49	1.4%
Tianjin	1894	26	1.4%
Anhui	11,095	145	1.4%
Sichuan	15,946	204	1.4%
Zhejiang	9474	121	1.4%
Fujian	5181	66	1.4%
Ningxia Hui Autonomous Region	944	12	1.4%
Jilin	4170	52	1.2%
Hunan	11,775	136	1.1%
Chongqing	6156	69	1.1%
Inner Mongolia Autonomous Region	3306	37	1.1%
Henan	14,520	162	1.1%
Liaoning	8480	93	1.1%
Jiangxi	6147	67	1.1%
Heilongjiang	5551	59	1.1%
Yunnan	6605	65	1.0%
Shandong	17,564	154	0.9%
Hebei	10,608	93	0.9%
Shaanxi	5706	48	0.8%
Guizhou	5660	45	0.8%
Guangdong	13,249	101	0.8%
Shanxi	5211	39	0.7%
Qinghai	951	6	0.6%
Guangxi Zhuang Autonomous Region	8085	49	0.6%
Gansu	3324	20	0.6%
Hainan	1426	6	0.4%
Tibet Autonomous Region	923	2	0.2%
Total	212,578	2463	1.2%

### Analysis of factors related to malignant tumor prevalence in the elderly

The differences in malignant tumor prevalence among the elderly population based on various factors are displayed in Table 2. The study found that urban residents had a higher prevalence of malignant tumors compared to rural elderly individuals (1.39% and 0.88%, *p* < 0.001). Elderly individuals aged 80 and above had a lower prevalence of malignant tumors compared to those aged 60–69 and 70–79 (0.95%, 1.17%, and 1.19%, *p* = 0.002). The prevalence of malignant tumors was lower among elderly individuals living alone compared to those not living alone (0.89% and 1.19%, *p* < 0.001). Elderly individuals with medical insurance had a higher prevalence of malignant tumors compared to those without medical insurance (1.15% and 0.51%, *p* = 0.008). Elderly individuals engaged in paid work had a lower prevalence of malignant tumors compared to those without income (0.4% and 1.2%, *p* < 0.001). The prevalence of malignant tumors was lower among elderly individuals involved in public welfare activities compared to those not involved (1.07% and 1.21%, *p* = 0.002). Additionally, malignant tumor prevalence among the elderly was significantly associated with marital status (*p* < 0.001), alcohol consumption (*p* = 0.005), exercise habits (*p* = 0.018), educational level (*p* < 0.001), participation in public welfare activities (*p* = 0.002), and economic status (*p* < 0.001). There was no significant difference in malignant tumor prevalence based on sex, smoking status, presence of other elderly individuals requiring care in the household, experience of external abuse, or engagement in mental and cultural activities.

**Table 2:** Differences in malignant tumor prevalence based on various factors

Factor	The overall number of people	People without malignant tumors	People with malignant tumors	*P* value
**urban and rural**				0.000
000.00urban	111,940	110,385(98.61%)	1555(1.39%)	
rural	103,101	102,193(99.12%)	908(0.88%)	
**age**				
60–69	121,636	120,210(98.83%)	1426(1.17%)	***0.002***
70–79	63,891	63,133(98.81%)	758(1.19%)	
> 80	29,514	29,235(99.05%)	279(0.95%)	
**sex**				
female	112,349	111,100(98.89%)	1249(1.11%)	*0.125*
male	102,692	101,478(98.82%)	1214(1.18%)	
**marital status**				***0.000***
married	155,973	154,058(98.77%)	1915(1.23%)	
widowed	54,142	53,641(99.07%)	501(0.93%)	
divorce	1795	1766(98.38%)	29(1.62%)	
Single/never married	3131	3113(99.43%)	18(0.57%)	
**living Alone**				***0.000***
no	186,118	183,911(98.81%)	2207(1.19%)	
yes	28,923	28,667(99.11%)	256(0.89%)	
**smoke**				*0.490*
no	14,533	14,358(98.80%)	175(1.20%)	
yes	200,508	198,220(98.86%)	2288(1.14%)	
**alcohol drinking**				***0.005***
don’t drink or occasionally	211,936	209,489(98.85%)	2447(1.15%)	
1–2 times/week	849	848(99.88%)	1(0.12%)	
> 2 times/week	1970	1957(99.34%)	13(0.66%)	
frequently	286	284(99.30%)	2(0.70%)	
**exercise**				***0.018***
never	105,213	104,055(98.90%)	1158(1.10%)	
Less than 1 time	9453	9328(98.68%)	125(1.32%)	
1–2 times	27,582	27,289(98.94%)	293(1.06%)	
3–5 times	26,339	2603(98.86%)	300(1.14%)	
> 5 times	46,454	45,867(98.74%)	587(1.26%)	
**medical security**				***0.008***
no	1960	1950(99.49%)	10(0.51%)	
yes	213,081	210,628(98.85%)	2453(1.15%)	
**other elderly people in the family who need care**				*0.329*
no	189,767	187,609(98.86%)	2158(1.14%)	
yes	25,274	24,969(98.79%)	305(1.21%)	
**income**				***0.000***
no	193,528	191,158(98.78%)	2370(1.22%)	
yes	21,513	21,420(99.57%)	93(0.43%)	
**degree of education**				***0.000***
never went to school	63,102	62,558(99.14%)	544(0.86%)	
primary school	89,059	88,126(98.95%)	933(1.05%)	
junior school	40,508	39,938(98.59%)	570(1.41%)	
high /secondary /vocational high school	15,087	14,837(98.34%)	250(1.66%)	
junior college	4269	4175(97.80%)	94(2.20%)	
bachelor degree or above	2322	2256(97.16%)	66(2.84%)	
**participation in public welfare activities**				***0.002***
nonparticipation	117,267	115,847(98.79%)	1420(1.21%)	
participation	97,774	96,731(98.93%)	1043(1.07%)	
**economic conditions**				***0.000***
very rich	2738	2711(99.01%)	27(0.99%)	
better rich	31,721	31,471(99.21%)	250(0.79%)	
basically enough	126,650	125,450(99.05%)	1200(0.95%)	
poorer	45,135	44,427(98.43%)	708(1.57%)	
very poor	8797	8519(96.84%)	278(3.16%)	
**external abuse**				*0.177*
no	9623	9499(98.71%)	124(1.29%)	
yes	205,418	203,079(98.86%)	2339(1.14%)	
**spiritual and cultural life**				*0.736*
no	198,149	195,875(98.85%)	2274(1.15%)	
yes	16,892	16,703(98.88%)	189 (1.12%)	

**Fig. 2:**
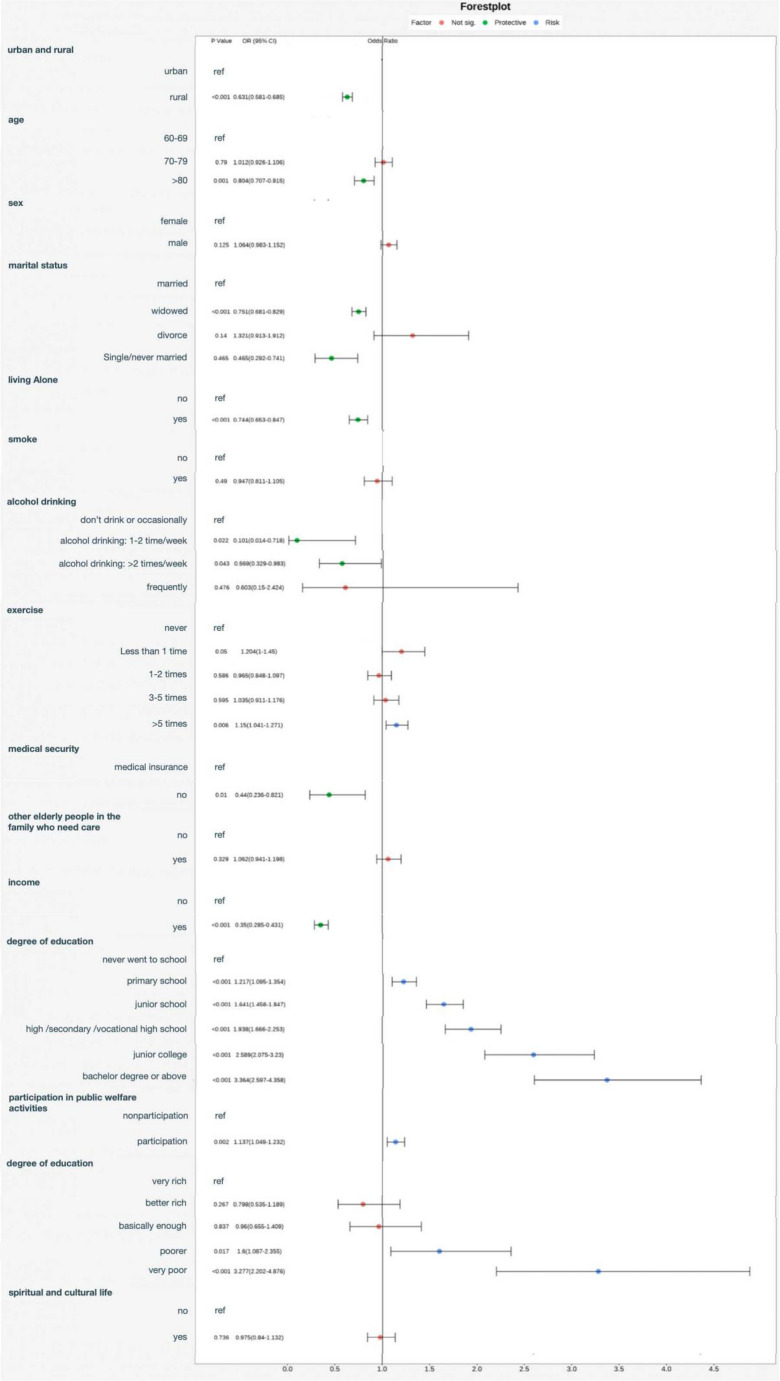
The results of the single-factor analysis of malignant tumor prevalence risk in the elderly population in China

The results of the single-factor logistic regression analysis are shown in Fig. 2. The study revealed significant associations between the prevalence of malignant tumors and factors such as household registration, age, marital status, living alone, alcohol consumption, exercise habits, medical insurance coverage, education level, participation in public welfare activities, engagement in paid work, and economic status. Among these factors, individuals with a rural household registration had a lower prevalence of malignant tumors compared to those with an urban household registration (OR = 0.631, 95% CI 0.581–0.685, *p* < 0.001). Older adults aged 80 years and above had a lower prevalence of malignant tumors compared to those aged 60–69 years (OR = 0.804, 95% CI 0.707–0.915, *p* = 0.001). Widowed individuals had a lower prevalence of malignant tumors compared to other groups (OR = 0.751, 95% CI 0.681–0.829, *p* < 0.001). The prevalence of malignant tumors was lower among individuals living alone compared to those living with others (OR = 0.744, 95% CI 0.653–0.847, *p* < 0.001). Individuals who consumed alcohol 1–2 times per week had a lower prevalence of malignant tumors compared to other groups (OR = 0.101, 95% CI 0.014–0.718, *p* = 0.022). The prevalence of malignant tumors was higher among individuals who exercised more than 6 times per week compared to those who exercised < 6 times or did not exercise (OR = 1.150, 95% CI 1.041–1.271, *p* = 0.006). Individuals without medical insurance coverage had a lower prevalence of malignant tumors compared to those with medical insurance coverage (OR = 0.440, 95% CI 0.236–0.821, *p* = 0.01). The prevalence of malignant tumors was lower among individuals engaged in paid activities compared to those without paid activities (OR = 0.350, 95% CI 0.285–0.431, *p* < 0.001). Furthermore, the higher the education level, the higher the prevalence of malignant tumors, with the highest prevalence observed among individuals with a bachelor’s degree or higher (OR = 3.364, 95% CI 2.597–4.358, *p* < 0.001). Similarly, individuals with poorer economic status had a higher prevalence of malignant tumors, with the highest risk observed among those facing extreme financial difficulties (OR = 3.277, 95% CI 2.202–4.876, *p* < 0.001).

**Fig. 3:**
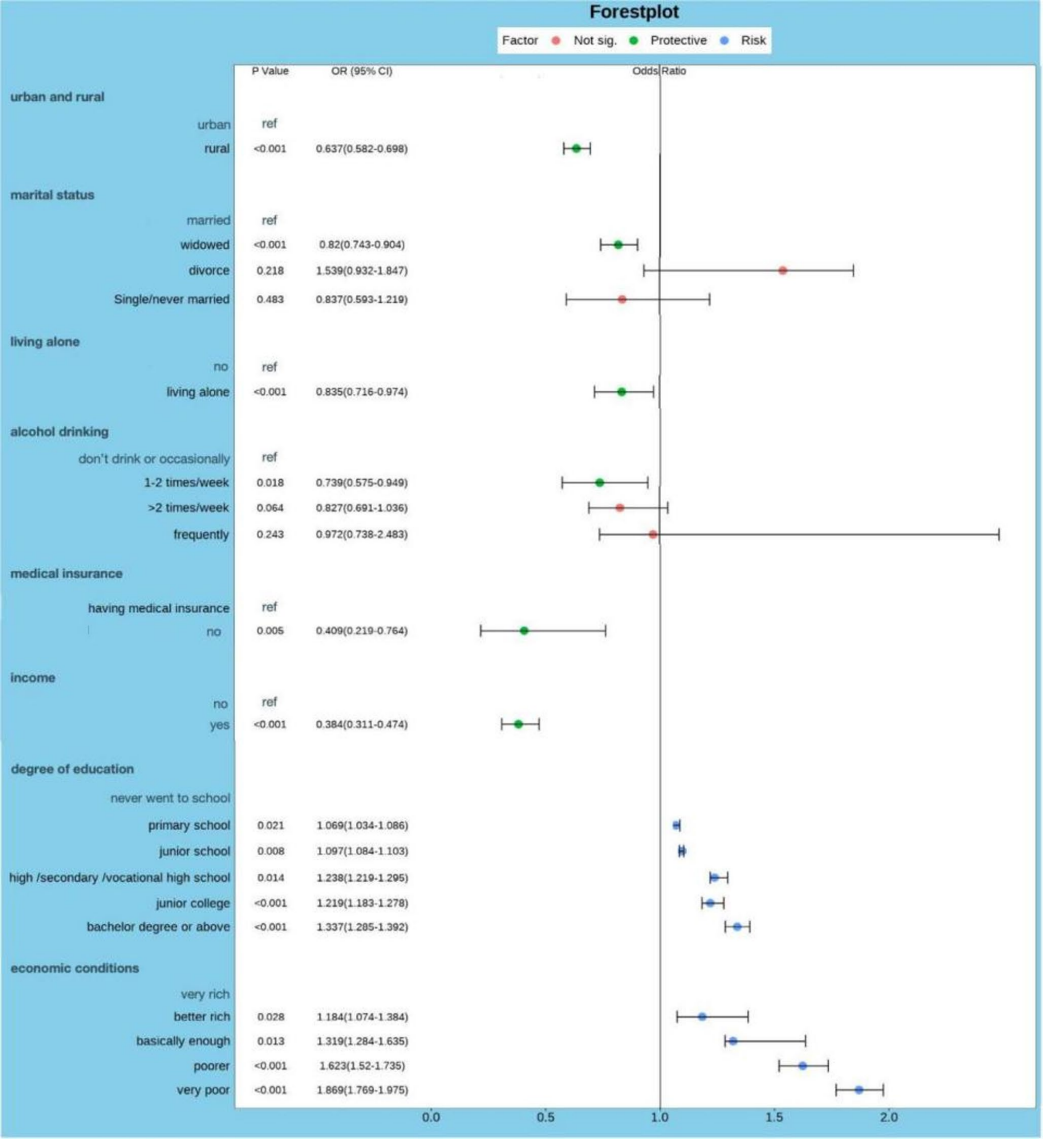
The results of the multivariable analysis of malignant tumor prevalence risk in the elderly population in China

Multivariable logistic regression was used to eliminate the influence of other confounding factors and determine the association between factors and malignant tumor prevalence. Multivariable logistic regression analysis was performed on the test results with *p* < 0.05 from the single-factor analysis. The results are shown in Fig. 3, indicating that household registration, marital status, living alone, alcohol consumption, medical insurance coverage, income status, education level, and economic status are independently associated with malignant tumor prevalence. Among them, rural household registration (OR = 0.637, 95% CI 0.582–0.698, *p* < 0.001), being widowed (OR = 0.820, 95% CI 0.743–0.904, *p* < 0.001), living alone (OR = 0.835, 95% CI 0.716–0.974, *p* < 0.001), moderate alcohol consumption (OR = 0.739, 95% CI 0.575–0.949, *p* = 0.018), not having medical insurance coverage (OR = 0.409, 95% CI 0.219–0.764, *p* = 0.005), and having income (OR = 0.384, 95% CI 0.311–0.474, *p* < 0.001) were associated with a lower prevalence of malignant tumors. On the other hand, higher education level (OR = 1.337, 95% CI 1.285–1.392, *p* < 0.001) and poorer economic status (OR = 1.869, 95% CI 1.769–1.975, *p* < 0.001) were associated with a higher prevalence of malignant tumors in the elderly population.

### Stratified analysis based on urban-rural, sex, and age

To control for confounding factors, a stratified analysis was conducted based on urban-rural, sex, and age to examine the association between relevant variables and malignant tumor prevalence within each stratum.

According to the participants’ urban-rural status, they were divided into two groups: urban residents (111,940 individuals) with a malignant tumor prevalence rate of 1.4% and rural residents (103,101 individuals) with a malignant tumor prevalence rate of 0.9%. As shown in Table S3, significant differences in malignant tumor prevalence were observed among both urban and rural residents in terms of marital status, living alone, income status, education level, and economic status. Malignant tumor prevalence among rural residents varied significantly across different age groups (*p* < 0.001), while no age differences were found among urban residents (*p* = 0.648). Among urban residents, significant differences in malignant tumor prevalence were observed based on alcohol consumption (*p* = 0.015), medical insurance coverage (*p* = 0.032), and participation in charity activities (*p* < 0.001), while no statistical differences were observed among rural residents.

After stratifying by sex, among 112,349 female participants, the prevalence rate of malignant tumors was 1.1%, while among 102,692 male participants, the prevalence rate was 1.2%. Both male and female residents showed significant differences in malignant tumor prevalence based on urban-rural status, age, marital status, presence or absence of income, educational level, and economic status (Table S4). Among female residents, significant differences in malignant tumor prevalence were observed based on exercise frequency (*p* = 0.03), presence or absence of medical insurance (*p* = 0.008), and presence or absence of other elderly individuals requiring care in the household (*p* = 0.001). However, no significant differences were observed among male residents in these aspects. Male residents showed significant differences in malignant tumor prevalence based on living alone (*p* < 0.001) and alcohol consumption (*p* = 0.004).

After stratifying by age, there were a total of 121,636 individuals aged 60–69 years, with a malignant tumor prevalence rate of 1.2%. There were 63,891 individuals aged 70–79 years, with a malignant tumor prevalence rate of 1.2%. Lastly, there were 29,514 individuals aged 80 years and above, with a malignant tumor prevalence rate of 0.9%. Significant differences in malignant tumor prevalence were observed among the three age subgroups in terms of urban-rural status, sex, marital status, educational level, participation in public welfare activities, and economic status (Table S5). Among individuals aged 60–69 and 70–79 years, there were significant differences in malignant tumor prevalence between those living alone and those not living alone (*p* = 0.038 and *p* = 0.001, respectively). Among individuals aged 60–69 years, there were significant differences in malignant tumor prevalence based on different drinking habits (*p* = 0.005), and those with income had a significantly lower prevalence compared to those without income (0.4% and 1.3%, respectively, *p* < 0.001). Among individuals aged 80 years and above, the prevalence rate of malignant tumors was significantly higher when there were other elderly individuals requiring care in the household (*p* = 0.012), when there was external abuse (*p* = 0.044), and when there was engagement in cultural and intellectual activities (*p* = 0.041).

## Discussion

Our nationally representative study utilized stratified multi-stage probability proportionate to size sampling (PPS) across all 31 provincial-level administrative divisions, demonstrating high consistency with 2015 National Bureau of Statistics data in key demographic indicators: age structure (14.3% vs. 14.5% aged ≥ 65), sex ratio (48.7% vs. 48.9% female), and urbanization rate (57.1% vs. 56.1%). Recent data from the Global Burden of Disease (GBD) study [20] report show age-specific prevalence rates increasing over the past two decades in older age groups (50–69 and 70 + years), reflecting the growing burden of cancer in China’s aging population. Our study, focusing on individuals aged 60 and above, provides complementary epidemiological insights within this demographic, contributing valuable cross-sectional data despite differences in time frame and methodology. Notably, the prevalence rates observed in our 2015 dataset are similar to those reported in the GBD study for the year 2000, which may reflect the temporal progression of cancer prevalence in this population. However, differences in study design, population age grouping, and data collection methods may contribute to variation in reported prevalence estimates, and these factors should be considered when interpreting and comparing results across studies.

Based on this robust sampling framework, our findings reveal that urban elderly residents have significantly higher overall malignant tumor prevalence rates compared to their rural counterparts. This urban-rural disparity aligns with national tumor registration data from 501 registries, which reported age-standardized incidence rates of 304.96/100,000 in urban areas versus 261.40/100,000 in rural areas [17]. The observed geographical variations in cancer incidence patterns reinforce the necessity for region-specific prevention strategies. Similar distribution patterns of malignant tumor incidence rates were found in specific regions. According to the tumor incidence data from the Chronic Disease Detection Information Management System in Shaoxing City, Zhejiang Province in 2012, the incidence rate of malignant tumors in urban areas (364.85/100,000) was higher than that in rural areas (277.86/100,000) [21]. Based on the prevalence and mortality data of malignant tumors collected by the Shanghai Malignant Tumor Case Reporting and Registration System, as of December 31, 2016, there were a total of 399,027 surviving cancer patients in Shanghai, with a prevalence rate of 2.77%. The prevalence rate in urban areas was 3.07%, significantly higher than the prevalence rate in rural areas [22].

There may be various reasons for the urban-rural disparity in malignant tumors. In terms of the etiological factors of malignant tumors, environmental pollution and metabolic syndrome may contribute to the higher prevalence of malignant tumors. In urban environments, industrial pollution such as air and water pollution could be possible reasons for the increased incidence of certain malignant tumors [23]. Additionally, the higher prevalence of metabolic syndrome among urban residents may be associated with a higher incidence rate of certain malignant tumors to some extent [24]. Furthermore, compared to rural areas, urban residents generally have better medical conditions and higher health awareness, which could lead to increased detection of malignant tumors. While acknowledging the limitations of tumor-type aggregation, our socioeconomic profile (OR = 3.364 [2.597–4.358] for higher education) highlights potential healthcare disparities, where urban educated populations show higher cancer prevalence rates, which may reflect differences in healthcare access, including cancer screening services. However, it is important to note that this correlation does not directly imply better screening access, as the observed association could be influenced by recall bias and other confounding factors. We recommend that future studies utilize more comprehensive datasets, including subtype-specific registries, and further investigate the role of socioeconomic factors in cancer prevention and control strategies.

Lack of social contact is associated with an increased risk of cancer-related mortality, but there is limited research on whether living alone increases the prevalence rate of cancer. Elovainio et al. conducted a study using data from the Finnish Cancer Registry to examine the relationship between living alone and the incidence rates of eight common cancers. The study found an association between living alone and a higher incidence rate of lung cancer but a lower incidence rate of prostate cancer and melanoma [25]. In our study, living alone was associated with a lower overall prevalence of malignant tumors. After stratifying by sex, this association was only found in males, while no difference was observed among females, which requires further research to clarify.

The impact of marital status on cancer prevalence rates is still controversial. A systematic evaluation of the association between marital status and cancer risk was conducted in an Italian study using data from a case-control study conducted between 1983 and 2001. The study found that unmarried individuals had a significantly higher risk of developing oral and pharyngeal cancer compared to married individuals, while the risk of colon cancer, liver cancer, bladder cancer, kidney cancer, and thyroid cancer was lower. Divorced or widowed participants had an excessive prevalence [26]. In a systematic review by Buja et al. in 2018, which included 18 studies, unmarried patients (single, divorced/separated, and widowed) were found to have a higher risk of higher stage (stage II and above) malignancies in many tumor sites (lung, colon, pancreas, head and neck, esophagus, liver intrahepatic bile ducts, ovaries) [27]. In 2023, Kaja Krajc et al. conducted a systematic review of 67 studies to explore the impact of marital status on cancer survival. The meta-analysis showed that married patients had higher overall survival rates and cancer-specific survival rates compared to unmarried patients (single, divorced/separated, and widowed). However, their study grouped unmarried patients into one category, and the differences between these subgroups could not be further analyzed due to the heterogeneity of the included studies [28]. In our study, the overall prevalence of malignant tumors was highest in divorced elderly individuals and lowest in never-married individuals. Among women, widows had the highest prevalence rate, while among men, the prevalence rate was lower in never-married and widowed individuals.

Yoon-Jung Choi et al. [29] conducted a meta-analysis in 2018, including 60 cohort studies from 135 articles, to analyze the impact of light alcohol consumption on cancer prevalence rates. The meta-analysis showed that light drinking (≤ 0.5 drinks/day) or moderate drinking (≤ 1 drink/day) was not associated with the prevalence of most cancers. However, moderate drinking was significantly associated with a higher prevalence of colorectal cancer in men and breast cancer in women, while it was associated with a lower prevalence of hematological malignancies in both women and men. In our study, regardless of sex subgroup, the prevalence of tumors was lowest among those who drank moderately (1–2 times per week). The prevalence of malignant tumors increased with increasing alcohol consumption in male patients. However, because our study was based on self-reported questionnaire surveys, the patients’ alcohol consumption could not be objectively assessed, and the discussion did not include specific types of cancer, which may introduce certain biases. Additionally, most previous large cohort studies were conducted in Western countries, with fewer large-scale cohort studies from Asia. Large cohort studies from China have shown that alcohol consumption does not affect the prevalence of colorectal cancer, stomach cancer, and esophageal cancer in both men and women, which contradicts the findings of Yoon-Jung Choi et al., suggesting that further research is needed to investigate the impact of ethnic factors on the results [30–32].

According to the literature, the relationship between education level and the prevalence of malignant tumors is complex and varies across different types of cancer. Many studies suggest that a lower education level is significantly associated with higher incidence rates of certain malignancies that are closely related to lifestyle and dietary habits. This relationship can also influence prevalence, as higher incidence rates may lead to a higher prevalence of these cancers in the population. Individuals with higher education levels are often associated with healthier lifestyles, dietary habits, and greater health awareness, which may facilitate earlier cancer detection and improved access to medical care. This association is supported by studies showing that individuals with higher socio-economic positions (SEP) tend to have better awareness of cancer risk factors and symptoms, leading to more proactive healthcare-seeking behaviors and better outcomes in cancer detection [33]. A study conducted in Norway found that individuals with the highest education level had higher incidence rates of melanoma in males, testicular cancer in males, prostate cancer in males, and breast cancer in females. Compared to primary education, men and women with university or college education had a lower incidence of lung cancer, which could be indicative of better detection and healthcare access, possibly leading to a higher prevalence in more educated populations [34]. Another study from Iceland revealed a significant association between education level and cancer prevalence. Among academically educated men, the standardized incidence rates of prostate cancer and melanoma were higher, while the rates of lung cancer and stomach cancer were lower. Women with higher education had an increased prevalence of breast cancer and a decreased prevalence of lung cancer. Additionally, an elevated education level was associated with a decreased prevalence of cervical cancer [35]. Mouw et al. found that compared to men with a high school education, men with the lowest education level had a higher incidence of esophageal cancer, head and neck cancer, gastric cancer, colon cancer, rectal cancer, liver cancer, lung cancer, pleural cancer, bladder cancer, and smoking-related composite cancers. In contrast, a lower education level was associated with a decreased prevalence of melanoma and localized prostate cancer. Women with the lowest education level had a higher prevalence of colon cancer, lung cancer, renal cancer, and smoking-related composite cancers, but a lower prevalence of melanoma, endometrial cancer, and invasive breast cancer [36]. In our study, we found a positive correlation between the overall prevalence of malignant tumors in elderly individuals and their education level. As education level increased, the prevalence rate of malignant tumors also increased. This association may be explained by better health awareness and earlier cancer detection in individuals with higher education. However, further research is needed to analyze the specific types of malignant tumors and to validate the relationship between education level and cancer prevalence across different regions and populations.

Our research findings indicate that the prevalence of malignant tumors among older adults in China gradually decreases with improvements in the economic situation. The elderly population facing extremely difficult economic conditions has the highest risk of malignant tumor occurrence, with a risk ratio of 3.277 times compared to the affluent population (OR = 3.277, 95% CI 2.202–4.876, *p* < 0.001). This conclusion aligns with findings from other studies. Lagergren et al. found that the incidence rate ratio (IRR) for esophageal and gastric cancers, comparing participants from the highest and lowest income levels (top 20% and bottom 20% of income), was 0.74 (95% CI, 0.70–0.79) for males and 0.83 (95% CI, 0.76–0.91) for females. Regardless of sex, individuals with higher incomes had lower incidence rates of esophageal and gastric cancers [37]. Larsen et al. [34] reported that, in males, the IRR for malignant tumors such as lung cancer, esophageal cancer, liver cancer, and Hodgkin’s lymphoma significantly decreased in the higher-income group compared to the middle-income group. However, there was no significant difference in the risk of malignant tumors among different income groups in females. These associations may be attributed to better economic conditions, improved lifestyles, health awareness, and superior healthcare services.

This study provides a comprehensive assessment of the overall prevalence and geographical distribution of malignant tumors among the elderly population in China, based on a large, nationally representative sample. Although detailed information on specific cancer types and precise diagnosis time intervals was not available, limiting subtype-specific analyses, our findings contribute valuable insights into the burden and distribution patterns of malignant tumors within China’s aging population. We examined various factors, including sex, age, living conditions, and socio-economic behaviors, that are potentially associated with malignant tumor prevalence. Amid the aging population and rising prevalence of malignant tumors, this study further explores the prevalence and distribution of malignant tumors among the elderly across China, offering additional insights to inform future public health strategies. Given ongoing demographic shifts and the increasing burden of malignant tumors, addressing disparities between urban and rural areas, as well as regional imbalances, remains a critical challenge. Therefore, it is essential to enhance public awareness, support interventions aimed at modifiable risk factors, and develop comprehensive strategies to mitigate the growing impact of malignant tumors in China’s elderly population.

## Limitations

This study has some limitations. Firstly, the specific pathological types and treatment information of malignant tumors were not recorded in the survey. Therefore, the research conclusions can only be analyzed regarding the overall prevalence of malignant tumors. Since the etiology and risk factors for different malignant tumors vary, each tumor may have its own specific epidemiological characteristics, and this study cannot obtain epidemiological data on specific tumor types for further analysis. Secondly, this study is a cross-sectional study, and the analysis of potential risk factors is based on correlation analysis, not causal relationship analysis. Therefore, causal conclusions cannot be drawn. Thirdly, similar to other large-scale population surveys, the determination of malignant tumors in the survey mainly relies on standardized questionnaires. Given the emotional and psychological support provided by family members and healthcare professionals, some elderly individuals may be uninformed about their own tumor conditions, leading to potential information bias and possibly underestimating the actual tumor prevalence rate within the elderly population. Fourthly, the variable settings are inadequate, for example, common chronic diseases such as hypertension, diabetes, and cardiovascular diseases were not included as coexisting conditions, making it impossible to analyze the impact of such factors on the occurrence of malignant tumors. Fifthly, this study utilized cross-sectional data from 2015, which may not fully capture temporal trends in cancer prevalence over the past decade. However, recent evidence [38] indicates that urban-rural disparities in cancer incidence and healthcare access persist in China, suggesting the ongoing relevance of our findings. Finally, our data related to alcohol consumption was qualitative rather than quantitative, limiting our ability to assess the specific influence of alcohol consumption levels on the occurrence of malignant tumors.

## Conclusions

To the best of our knowledge, this study represents the most complete and up-to-date report on the overall prevalence and distribution of malignancies among the elderly over 60 years of age in China by urban-rural region, sex, and age. Compared with the elderly aged 60–69 and those over 80 years old, the prevalence of malignant tumors in the 70–79 age group is the highest. The prevalence of malignant tumors in urban elderly is higher than that in rural residents. There was no significant difference in the overall prevalence of malignant tumors between sexs. Living in cities, not living alone, higher education levels, and poor economic conditions may be independent risk factors for malignant tumors in the elderly, while widowhood and moderate alcohol consumption may be protective factors. More detailed and more content questionnaires and surveys with larger sample sizes are necessary to ensure the research conclusions.

## Electronic supplementary material

Below is the link to the electronic supplementary material.

Supplementary Material 1: Table S3: Malignant Tumor Prevalence Rate Stratified by Urban-Rural Status.Table S4: Malignant Tumor Prevalence Rates Stratified by sex. Table S5: Prevalence of Malignant Tumors Stratified by Age. STROBE checklist.

## Data Availability

Data is provided within the manuscript or supplementary information files.
