# Supplementary material for: Reported prevalence and risk factors of malignant tumors in the elderly population in China: a nationwide cross-sectional study
Source: Aging Clin Exp Res. 2025 Jun 23;37(1):194. doi: 10.1007/s40520-025-03109-1 (PMC12185627; doi:10.1007/s40520-025-03109-1)
Supplement: Supplementary file 1 — Supplementary Material 1: Table S3: Malignant Tumor Prevalence Rate Stratified by Urban-Rural Status.Table S4: Malignant Tumor Prevalence Rates Stratified by sex. Table S5: Prevalence of Malignant Tumors Stratified by Age. STROBE checklist. [file 40520_2025_3109_MOESM1_ESM.docx]

**Supplementary Materials**

**Table S3: Malignant Tumor Prevalence Rate Stratified by Urban-Rural Status**

| **Factor** | **Urban** | | | | | | **Rural** | | | | | | | | | |
| --- | --- | --- | --- | --- | --- | --- | --- | --- | --- | --- | --- | --- | --- | --- | --- | --- |
|  | **Overall number of people** | **People without malignant tumors** | | **People with malignant tumors** | ***P value*** | | **Overall number of people** | | | **People without malignant tumors** | | | **People with malignant tumors** | | ***P value*** | |
| **age** |  |  | |  |  | |  | | |  | | |  | |  | |
| 60-69 | 62046 | 61191(98.62%) | | 855(1.38%) | *0.648* | | 59590 | | | 59019(99.04%) | | | 571(0.96%) | | ***0.000*** | |
| 70-79 | 33458 | 32978(98.57%) | | 480(1.43%) |  | | 30433 | | | 30155(99.09%) | | | 278(0.91%) | |  | |
| >80 | 16436 | 16216(98.66%) | | 220(1.34%) |  | | 13078 | | | 13019(99.55%) | | | 59(0.45%) | |  | |
| **sex** |  |  |  | | |  | |  |  | |  | | |  | |  |
| female | 59670 | 58861(98.64%) | | 809(1.36%) | *0.308* | | 52679 | | | 52239(99.16%) | | | 440(0.84%) | | *0.110* | |
| male | 52270 | 51524(98.57%) | | 746(1.43%) |  | | 50422 | | | 49954(99.07%) | | | 468(0.93%) | |  | |
| **Marital status** |  |  |  | | |  | |  |  | |  | | |  | |  |
| married | 82520 | 81329(98.56%) | | 1191(1.44%) | ***0.010*** | | 73453 | | | 72729(99.01%) | | | 724(0.99%) | | ***0.000*** | |
| widowed | 27175 | 26848(98.80%) | | 327(1.20%) |  | | 26967 | | | 26793(99.35%) | | | 174(0.65%) | |  | |
| divorce | 1299 | 1274(98.08%) | | 25(1.92%) |  | | 496 | | | 492(99.19%) | | | 4(0.81%) | |  | |
| Single/never married | 946 | 934(98.73%) | | 12(1.27%) |  | | 2185 | | | 2179(99.73%) | | | 6(0.27%) | |  | |
| **living alone** |  |  |  | | |  | |  |  | |  | | |  | |  |
| no | 98138 | 96748(98.58%) | | 1390(1.42%) | ***0.038*** | | 87980 | | | 87163(99.07%) | | | 817(0.93%) | | ***0.000*** | |
| yes | 13802 | 13637(98.80%) | | 165(1.20%) |  | | 15121 | | | 15030(99.40%) | | | 91(0.60%) | |  | |
| **smoke** |  |  |  | | |  | |  |  | |  | | |  | |  |
| no | 7860 | 7748(98.58%) | | 112(1.42%) | *0.779* | | 6673 | | | 6610(99.06%) | | | 63(0.94%) | | *0.566* | |
| yes | 104080 | 102637(98.61%) | | 1443(1.39%) |  | | 96428 | | | 95583(99.12%) | | | 845(0.88%) | |  | |
| **alcohol drinking** |  |  |  | | |  | |  |  | |  | | |  | |  |
| don’t drink or occasionally | 110488 | 108939(98.60%) | | 1549(1.40%) | ***0.015*** | | 101448 | | | 100550(99.11%) | | | 898(0.89%) | | *0.251* | |
| 1-2 times/week | 406 | 405(99.75%) | | 1(0.25%) |  | | 443 | | | 443(100.00%) | | | 0(0.00%) | |  | |
| >2 times/week | 924 | 920(99.57%) | | 4(0.43%) |  | | 1046 | | | 1037(99.14%) | | | 9(0.86%) | |  | |
| frequently | 122 | 121(99.18%) | | 1(0.82%) |  | | 164 | | | 163(99.39%) | | | 1(0.61%) | |  | |
| **exercise** |  |  |  | | | | |  |  | |  | | |  | |  |
| never | 39167 | 38588(98.52%) | | 579(1.48%) | *0.152* | | 66046 | | | 65467(99.12%) | | | 579(0.88%) | | *0.501* | |
| less than 1 time | 5350 | 5155(96.36%) | | 195(3.64%) |  | | 4216 | | | 4173(98.98%) | | | 43(1.02%) | |  | |
| 1-2 times | 15732 | 15537(98.76%) | | 195(1.24%) |  | | 11850 | | | 11752(99.17%) | | | 98(0.83%) | |  | |
| 3-5 times | 16914 | 16689(98.67%) | | 225(1.33%) |  | | 9425 | | | 9350(99.20%) | | | 75(0.80%) | |  | |
| >5 times | 34890 | 34416(98.64%) | | 474(1.36%) |  | | 11564 | | | 11451(99.02%) | | | 113(0.98%) | |  | |
| **medical security** |  |  |  | | | | |  |  | |  | | |  | |  |
| no | 999 | 993(99.40%) | | 6(0.60%) | ***0.032*** | | 961 | | | 957(99.58%) | | | 4(0.42%) | | *0.122* | |
| yes | 110941 | 109392(98.60%) | | 1549(1.40%) |  | | 102140 | | | 101236(99.11%) | | | 904(0.89%) | |  | |
| other elderly people in the family who need care | |  |  | | | | |  |  | |  | | |  | |  |
| no | 98332 | 96979(98.62%) | | 1353(1.38%) | *0.311* | | 91435 | | | 90630(99.12%) | | | 805(0.88%) | | *0.978* | |
| yes | 13608 | 13406(98.52%) | | 202(1.48%) |  | | 11666 | | | 11563(99.12%) | | | 103(0.88%) | |  | |
| **income** |  |  |  | | | | |  |  | |  | | |  | |  |
| no | 101195 | 99695(98.52%) | | 1500(1.48%) | ***0.000*** | | 92333 | | | 91463(99.06%) | | | 870(0.94%) | | ***0.000*** | |
| yes | 10745 | 10690(99.49%) | | 55(0.51%) |  | | 10768 | | | 10730(99.65%) | | | 38(0.35%) | |  | |
| **degree of education** |  |  |  | | | | |  |  | |  | | |  | |  |
| never went to school | 24585 | 24328(98.95%) | | 257(1.05%) | ***0.000*** | | 38517 | | | 38230(99.25%) | | | 287(0.75%) | | ***0.006*** | |
| primary school | 41104 | 40627(98.84%) | | 477(1.16%) |  | | 47955 | | | 47499(99.05%) | | | 456(0.95%) | |  | |
| junior school | 26763 | 26322(98.35%) | | 441(1.65%) |  | | 13745 | | | 13616(99.06%) | | | 129(0.94%) | |  | |
| high /secondary /vocational high school | 12684 | 12465(98.27%) | | 219(1.73%) |  | | 2403 | | | 2372(98.71%) | | | 31(1.29%) | |  | |
| junior college | 4185 | 4092(97.78%) | | 93(2.22%) |  | | 84 | | | 83(98.81%) | | | 1(1.19%) | |  | |
| bachelor degree or above | 2314 | 2248(97.15%) | | 66(2.85%) |  | | 8 | | | 8(100.00%) | | | 0(0.00%) | |  | |
| **participation in public welfare activities** |  |  |  | | | | |  | | | |  | | | |  |
| nonparticipation | 63629 | 62671(98.49%) | | 958(1.51%) | ***0.000*** | | 53638 | | | 53176(99.14%) | | | 462(0.86%) | | *0.488* | |
| participation | 48311 | 47714(98.76%) | | 597(1.24%) |  | | 49463 | | | 49017(99.10%) | | | 446(0.90%) | |  | |
| **economic conditions** |  |  |  | | | | |  | | | |  | | | |  |
| very rich | 1899 | 1874(98.68%) | | 25(1.32%) | ***0.000*** | | 839 | | | 837(99.76%) | | | 2(0.24%) | | ***0.000*** | |
| Better rich | 20582 | 20391(99.07%) | | 191(0.93%) |  | | 11139 | | | 11080(99.47%) | | | 59(0.53%) | |  | |
| basically enough | 68942 | 68106(98.79%) | | 836(1.21%) |  | | 57708 | | | 57344(99.37%) | | | 364(0.63%) | |  | |
| poorer | 17258 | 16890(97.87%) | | 36(2.13%) |  | | 27877 | | | 27537(98.78%) | | | 340(1.22%) | |  | |
| very poor | 3259 | 3124(95.86%) | | 135(4.14%) |  | | 5538 | | | 5395(97.42%) | | | 143(2.58%) | |  | |
| **external abuse** |  |  |  | | | | |  | | | |  | | | |  |
| no | 106931 | 105454(98.62%) | | 1477(1.38%) | *0.298* | | 98487 | | | 97625(99.12%) | | | 862(0.88%) | | *0.387* | |
| yes | 5009 | 4931(98.44%) | | 78(1.56%) |  | | 4614 | | | 4568(99.00%) | | | 46(1.00%) | |  | |
| **spiritual and cultural life** |  |  |  | | | | |  | | | |  | | | |  |
| no | 106108 | 104648(98.62%) | | 1460(1.38%) | *0.108* | | 92041 | | | 91227(99.12%) | | | 814(0.88%) | | *0.714* | |
| yes | 5832 | 5737(98.37%) | | 95(1.63%) |  | | 11060 | | | 10966(99.15%) | | | 94(0.85%) | |  | |

**Table S4: Malignant Tumor Prevalence Rates Stratified by sex**

| factor | **female** | | | | **male** | | | |
| --- | --- | --- | --- | --- | --- | --- | --- | --- |
|  | **Overall number of people** | **People without malignant tumors** | **People with malignant tumors** | ***P value*** | **Overall number of people** | **People without malignant tumors** | **People with malignant tumors** | ***P value*** |
| **urban and rural** |  |  |  |  |  |  |  |  |
| urban | 59670 | 58861(98.64%) | 809(1.36%) | ***0.000*** | 52270 | 51524(98.57%) | 746(1.43%) | ***0.000*** |
| rural | 52679 | 52239(99.16%) | 440(0.84%) |  | 50422 | 49954(99.07%) | 468(0.93%) |  |
| **age** |  |  |  |  |  |  |  |  |
| 60-69 | 61868 | 61099(98.76%) | 769(1.24%) | ***0.000*** | 59768 | 59111(98.90%) | 657(1.10%) | ***0.013*** |
| 70-79 | 33455 | 33097(98.93%) | 358(1.07%) |  | 30436 | 30036(98.69%) | 400(1.31%) |  |
| >80 | 17026 | 16904(99.28%) | 122(0.72%) |  | 12488 | 12331(98.74%) | 157(1.26%) |  |
| **marital status** |  |  |  |  |  |  |  |  |
| married | 72146 | 71272(98.79%) | 874(1.21%) | ***0.000*** | 83827 | 82786(98.76%) | 1041(1.24%) | ***0.000*** |
| widowed | 39323 | 38967(99.09%) | 356(0.91%) |  | 14819 | 14674(99.02%) | 145(0.98%) |  |
| divorce | 705 | 690(97.87%) | 15(2.13%) |  | 1090 | 1076(98.72%) | 14(1.28%) |  |
| Single/never married | 175 | 171(97.71%) | 4(2.29%) |  | 2956 | 2942(99.53%) | 14(0.47%) |  |
| **living alone** |  |  |  |  |  |  |  |  |
| no | 95008 | 93933(98.87%) | 1075(1.13%) | *0.139* | 91110 | 89978(98.76%) | 1132(1.24%) | ***0.000*** |
| yes | 17341 | 17167(99.00%) | 174(1.00%) |  | 11582 | 11500(99.29%) | 82(0.71%) |  |
| **smoke** |  |  |  |  |  |  |  |  |
| no | 10685 | 10551(98.75%) | 134(1.25%) | *0.140* | 3848 | 3807(98.93%) | 41(1.07%) | *0.495* |
| yes | 101664 | 100549(98.90%) | 1115(1.10%) |  | 98844 | 97671(98.81%) | 1173(1.19%) |  |
| **alcohol drinking** |  |  |  |  |  |  |  |  |
| don’t drink or occasionally | 112018 | 110772(98.89%) | 1246(1.11%) | *0.566* | 99918 | 98717(98.80%) | 1201(1.20%) | ***0.004*** |
| 1-2 times/week | 124 | 124(100.00%) | 0(0.00%) |  | 725 | 724(99.86%) | 1(0.14%) |  |
| >2 times/week | 187 | 184(98.40%) | 3(1.60%) |  | 1783 | 1773(99.44%) | 10(0.56%) |  |
| frequently | 20 | 20(100.00%) | 0(0.00%) |  | 266 | 264(99.25%) | 2(0.75%) |  |
| **exercise** |  |  |  |  |  |  |  |  |
| never | 57648 | 57036(98.94%) | 612(1.06%) | ***0.030*** | 47565 | 47019(98.85%) | 546(1.15%) | *0.495* |
| less than 1 time | 5195 | 5122(98.59%) | 73(1.41%) |  | 4258 | 4206(98.78%) | 52(1.22%) |  |
| 1-2 times | 14516 | 14365(98.96%) | 151(1.04%) |  | 13066 | 12924(98.91%) | 142(1.09%) |  |
| 3-5 times | 13407 | 13265(98.94%) | 142(1.06%) |  | 12932 | 12774(98.78%) | 158(1.22%) |  |
| >5 times | 21583 | 21312(98.74%) | 271(1.26%) |  | 24871 | 24555(98.73%) | 316(1.27%) |  |
| **medical security** |  |  |  |  |  |  |  |  |
| no | 111260 | 110014(98.88%) | 1246(1.12%) | ***0.008*** | 101821 | 100614(98.81%) | 1207(1.19%) | *0.299* |
| yes | 1089 | 1086(99.72%) | 3(0.28%) |  | 871 | 864(99.20%) | 7(0.80%) |  |
| **other elderly people in the family who need care** |  |  |  |  |  |  |  |  |
| no | 99801 | 98727(98.92%) | 1074(1.08%) | ***0.001*** | 89966 | 88882(98.80%) | 1084(1.20%) | *0.073* |
| yes | 12548 | 12373(98.61%) | 175(1.39%) |  | 12726 | 12596(98.98%) | 130(1.02%) |  |
| **income** |  |  |  |  |  |  |  |  |
| no | 105982 | 104764(98.85%) | 1218(1.15%) | ***0.000*** | 87546 | 86394(98.68%) | 1152(1.32%) | ***0.000*** |
| yes | 6367 | 6336(99.51%) | 31(0.49%) |  | 15146 | 15084(99.59%) | 62(0.41%) |  |
| **degree of education** |  |  |  |  |  |  |  |  |
| never went to school | 48250 | 47840(99.15%) | 410(0.85%) | ***0.000*** | 14852 | 14718(99.10%) | 134(0.90%) | ***0.000*** |
| primary school | 41965 | 41531(98.97%) | 434(1.03%) |  | 47094 | 46595(98.94%) | 499(1.06%) |  |
| junior school | 14376 | 14138(98.34%) | 238(1.66%) |  | 26132 | 25800(98.73%) | 332(1.27%) |  |
| high /secondary /vocational high school | 5539 | 5429(98.01%) | 110(1.99%) |  | 9548 | 9408(98.53%) | 140(1.47%) |  |
| junior college | 1237 | 1204(97.33%) | 33(2.67%) |  | 3032 | 2971(97.99%) | 61(2.01%) |  |
| bachelor degree or above | 626 | 604(96.49%) | 22(3.51%) |  | 1696 | 1652(97.41%) | 44(2.59%) |  |
| **participation in public welfare activities** |  |  |  |  |  |  |  |  |
| nonparticipation | 64600 | 63870(98.87%) | 730(1.13%) |  | 52667 | 51977(98.69%) | 690(1.31%) |  |
| participation | 47749 | 47230(98.91%) | 519(1.09%) |  | 50025 | 49501(98.95%) | 524(1.05%) |  |
| **economic conditions** |  |  |  |  |  |  |  |  |
| very rich | 1270 | 1258(99.06%) | 12(0.94%) | ***0.000*** | 1468 | 1453(98.98%) | 15(1.02%) | ***0.000*** |
| better rich | 15489 | 15362(99.18%) | 127(0.82%) |  | 16232 | 16109(99.24%) | 123(0.76%) |  |
| basically enough | 66499 | 65879(99.07%) | 620(0.93%) |  | 60151 | 59571(99.04%) | 580(0.96%) |  |
| poorer | 24385 | 24031(98.55%) | 354(1.45%) |  | 20750 | 20396(98.29%) | 354(1.71%) |  |
| very poor | 4706 | 4570(97.11%) | 136(2.89%) |  | 4091 | 3949(96.53%) | 142(3.47%) |  |
| **external abuse** |  |  |  |  |  |  |  |  |
| no | 4871 | 4805(98.65%) | 66(1.35%) | *0.098* | 4752 | 4694(98.78%) | 58(1.22%) | *0.802* |
| yes | 107478 | 106295(98.90%) | 1183(1.10%) |  | 97940 | 96784(98.82%) | 1156(1.18%) |  |
| **spiritual and cultural life** |  |  |  |  |  |  |  |  |
| no | 101497 | 100362(98.88%) | 1135(1.12%) | *0.522* | 96652 | 95513(98.82%) | 1139(1.18%) | *0.659* |
| yes | 10852 | 10738(98.95%) | 114(1.05%) |  | 6040 | 5965(98.76%) | 75(1.24%) |  |

**Table S5: Prevalence of Malignant Tumors Stratified by Age**

| **factor** | **60-69** | | | | **70-79** | | | | **>80** | | | |
| --- | --- | --- | --- | --- | --- | --- | --- | --- | --- | --- | --- | --- |
|  | **Overall number of people** | **People without malignant tumors** | **People with malignant tumors** | ***P value*** | **Overall number of people** | **People without malignant tumors** | **People with malignant tumors** | ***P value*** | **Overall number of people** | **People without malignant tumors** | **People with malignant tumors** | ***P value*** |
| **urban and rural** |  |  |  |  |  |  |  |  |  |  |  |  |
| urban | 62046 | 61191(98.62%) | 855(1.38%) | ***0.000*** | 33458 | 32978(98.57%) | 480(1.43%) | ***0.000*** | 16436 | 16216(98.66%) | 220(1.34%) | ***0.000*** |
| rural | 59590 | 59019(99.04%) | 571(0.96%) |  | 30433 | 30155(99.09%) | 278(0.91%) |  | 13078 | 13019(99.55%) | 59(0.45%) |  |
| **sex** |  |  |  |  |  |  |  |  |  |  |  |  |
| female | 61868 | 61099(98.76%) | 769(1.24%) | ***0.020*** | 33455 | 33097(98.93%) | 358(1.07%) | ***0.040*** | 17026 | 16904(99.28%) | 122(0.72%) | ***0.000*** |
| male | 59768 | 59111(98.90%) | 657(1.10%) |  | 30436 | 30036(98.69%) | 400(1.31%) |  | 12488 | 12331(98.74%) | 157(1.26%) |  |
| **marital status** |  |  |  |  |  |  |  |  |  |  |  |  |
| married | 102784 | 101550(98.80%) | 1234(1.20%) | ***0.003*** | 41904 | 41360(98.70%) | 544(1.30%) | ***0.000*** | 11285 | 11148(98.79%) | 137(1.21%) | ***0.000*** |
| widowed | 15502 | 15341(98.96%) | 161(1.04%) |  | 20712 | 20508(99.02%) | 204(0.98%) |  | 17928 | 17792(99.24%) | 136(0.76%) |  |
| divorce | 1389 | 1367(98.42%) | 22(1.58%) |  | 319 | 313(98.12%) | 6(1.88%) |  | 87 | 86(98.85%) | 1(1.15%) |  |
| Single/never married | 1961 | 1952(99.54%) | 9(0.46%) |  | 956 | 952(99.58%) | 4(0.42%) |  | 214 | 209(97.66%) | 5(2.34%) |  |
| **living alone** |  |  |  |  |  |  |  |  |  |  |  |  |
| no | 111068 | 109744(98.81%) | 1324(1.19%) | ***0.038*** | 52630 | 51972(98.75%) | 658(1.25%) | ***0.001*** | 22420 | 22195(99.00%) | 225(1.00%) | *0.066* |
| yes | 10568 | 10466（99.03%） | 102(0.97%) |  | 11261 | 11161(99.11%) | 100(0.89%) |  | 7094 | 7040(99.24%) | 54(0.76%) |  |
| **smoke** |  |  |  |  |  |  |  |  |  |  |  |  |
| no | 8046 | 7943(98.72%) | 103(1.28%) | *0.353* | 4176 | 4126(98.80%) | 50(1.20%) | *0.946* | 2311 | 2289(99.05%) | 22(0.95%) | *0.973* |
| yes | 113590 | 112267(98.84%) | 1323(1.16%) |  | 59715 | 59007(98.81%) | 708(1.19%) |  | 27203 | 26946(99.06%) | 257(0.94%) |  |
| **alcohol drinking** |  |  |  |  |  |  |  |  |  |  |  |  |
| don’t drink or occasionally | 119608 | 118189(98.81%) | 1419(1.19%) | ***0.005*** | 63105 | 62355(98.81%) | 750(1.19%) | *0.807* | 29223 | 28945(99.05%) | 278(0.95%) | *0.724* |
| 1-2 times/week | 577 | 577(100.00%) | 0(0.00%) |  | 207 | 206(99.52%) | 1(0.48%) |  | 65 | 65(100.00%) | 0(0.00%) |  |
| >2 times/week | 1259 | 1253(99.52%) | 6(0.48%) |  | 517 | 511(98.84%) | 6(1.16%) |  | 194 | 193(99.48%) | 1(0.52%) |  |
| frequently | 192 | 191(99.48%) | 1(0.52%) |  | 62 | 61(98.39%) | 1(1.61%) |  | 32 | 32(100.00%) | 0(0.00%) |  |
| **exercise** |  |  |  |  |  |  |  |  |  |  |  |  |
| never | 56217 | 55589(98.88%) | 628(1.12%) | *0.216* | 31334 | 30968(98.83%) | 366(1.17%) | *0.310* | 17662 | 17498(99.07%) | 164(0.93%) | *0.299* |
| less than 1 time | 5031 | 4968(98.75%) | 63(1.25%) |  | 2831 | 2788(98.48%) | 43(1.52%) |  | 1591 | 1572(98.81%) | 19(1.19%) |  |
| 1-2 times | 16387 | 16206(98.90%) | 181(1.10%) |  | 7993 | 7903(98.87%) | 90(1.13%) |  | 3202 | 3180(99.31%) | 22(0.69%) |  |
| 3-5 times | 16186 | 15990(98.79%) | 196(1.21%) |  | 7649 | 7568(98.94%) | 81(1.06%) |  | 2504 | 2481(99.08%) | 23(0.92%) |  |
| >5 times | 27815 | 27457(98.71%) | 358(1.29%) |  | 14084 | 13906(98.74%) | 178(1.26%) |  | 4555 | 4504(98.88%) | 51(1.12%) |  |
| **medical security** |  |  |  |  |  |  |  |  |  |  |  |  |
| no | 120561 | 119142(98.82%) | 1419(1.18%) | *0.111* | 626870 | 626114(99.88%) | 756(0.12%) | *0.089* | 29150 | 28872(99.05%) | 278(0.95%) | *0.183* |
| yes | 1075 | 1068(99.35%) | 7(0.65%) |  | 521 | 519(99.62%) | 2(0.38%) |  | 364 | 363(99.73%) | 1(0.27%) |  |
| **other elderly people in the family who need care** |  |  |  |  |  |  |  |  |  |  |  |  |
| no | 105264 | 104017(98.82%) | 1247(1.18%) | *0.313* | 57634 | 56965(98.84%) | 669(1.16%) | *0.069* | 26869 | 26627(99.10%) | 242(0.90%) | ***0.012*** |
| yes | 16372 | 16193(98.91%) | 179(1.09%) |  | 6257 | 6168(98.58%) | 89(1.42%) |  | 2645 | 2608(98.60%) | 37(1.40%) |  |
| **income** |  |  |  |  |  |  |  |  |  |  |  |  |
| no | 103190 | 101832(98.68%) | 1358(1.32%) | ***0.000*** | 61092 | 60357(98.80%) | 735(1.20%) | *0.068* | 29246 | 28969(99.05%) | 277(0.95%) | *0.735* |
| yes | 18446 | 18378(99.63%) | 68(0.37%) |  | 2799 | 2776(99.18%) | 23(0.82%) |  | 268 | 266(99.25%) | 2(0.75%) |  |
| **degree of education** |  |  |  |  |  |  |  |  |  |  |  |  |
| never went to school | 25854 | 25591(98.98%) | 263(1.02%) | ***0.000*** | 21283 | 21098(99.13%) | 185(0.87%) | ***0.000*** | 15965 | 15869(99.40%) | 96(0.60%) | ***0.000*** |
| primary school | 54435 | 53851(98.93%) | 584(1.07%) |  | 25772 | 25500(98.94%) | 272(1.06%) |  | 8852 | 8775(99.13%) | 77(0.87%) |  |
| junior school | 28354 | 27970(98.65%) | 384(1.35%) |  | 9867 | 9719(98.50%) | 148(1.50%) |  | 2287 | 2249(98.34%) | 38(1.66%) |  |
| high /secondary /vocational high school | 9276 | 9149(98.63%) | 127(1.37%) |  | 4462 | 4370(97.94%) | 92(2.06%) |  | 1349 | 1318(97.70%) | 31(2.30%) |  |
| junior college | 2486 | 2438(98.07%) | 48(1.93%) |  | 1270 | 1238(97.48%) | 32(2.52%) |  | 513 | 499(97.27%) | 14(2.73%) |  |
| bachelor degree or above | 851 | 836(98.24%) | 15(1.76%) |  | 1021 | 993(97.26%) | 28(2.74%) |  | 450 | 427(94.89%) | 23(5.11%) |  |
| **participation in public welfare activities** |  |  |  |  |  |  |  |  |  |  |  |  |
| nonparticipation | 59098 | 58366(98.76%) | 732(1.24%) | *0.037* | 36543 | 36082(98.74%) | 461(1.26%) | ***0.043*** | 21626 | 21399(98.95%) | 227(1.05%) | ***0.002*** |
| participation | 62538 | 61844(98.89%) | 694(1.11%) |  | 27348 | 27051(98.91%) | 297(1.09%) |  | 7888 | 7836(99.34%) | 52(0.66%) |  |
| **economic conditions** |  |  |  |  |  |  |  |  |  |  |  |  |
| very rich | 1540 | 1531(99.42%) | 9(0.58%) | ***0.000*** | 790 | 780(98.73%) | 10(1.27%) | ***0.000*** | 408 | 400(98.04%) | 8(1.96%) | ***0.013*** |
| better rich | 18817 | 18691(99.33%) | 126(0.67%) |  | 8716 | 8640(99.13%) | 76(0.87%) |  | 4188 | 4140(98.85%) | 48(1.15%) |  |
| basically enough | 72906 | 72229(99.07%) | 677(0.93%) |  | 36880 | 36498(98.96%) | 382(1.04%) |  | 16864 | 16723(99.16%) | 141(0.84%) |  |
| poorer | 23935 | 23501(98.19%) | 434(1.81%) |  | 14580 | 14367(98.54%) | 213(1.46%) |  | 6620 | 6559(99.08%) | 61(0.92%) |  |
| very poor | 4438 | 4258(95.94%) | 180(4.06%) |  | 2925 | 2848(97.37%) | 77(2.63%) |  | 1434 | 1413(98.54%) | 21(1.46%) |  |
| **external abuse** |  |  |  |  |  |  |  |  |  |  |  |  |
| no | 5565 | 5493(98.71%) | 72(1.29%) | *0.389* | 3019 | 2983(98.81%) | 36(1.19%) | *0.975* | 1039 | 1023(98.46%) | 16(1.54%) | ***0.044*** |
| yes | 116071 | 114717(98.83%) | 1354(1.17%) |  | 60872 | 60150(98.81%) | 722(1.19%) |  | 28475 | 28212(99.08%) | 263(0.92%) |  |
| **spiritual and cultural life** |  |  |  |  |  |  |  |  |  |  |  |  |
| no | 115773 | 114429(98.84%) | 1344(1.16%) | *0.099* | 58276 | 57587(98.82%) | 689(1.18%) | *0.758* | 24100 | 23859(99.00%) | 241(1.00%) | ***0.041*** |
| yes | 5863 | 5781(98.60%) | 82(1.40%) |  | 5615 | 5546(98.77%) | 69(1.23%) |  | 5414 | 5376(99.30%) | 38(0.70%) |  |

STROBE Statement—Checklist of items that should be included in reports of ***cross-sectional studies***

|  | Item No | Recommendation |
| --- | --- | --- |
| **Title and abstract** | 1 | (*a*) Indicate the study’s design with a commonly used term in the title or the abstract**✅ P1** |
|  |  | (*b*) Provide in the abstract an informative and balanced summary of what was done and what was found ✅**P1** |
| Introduction | | |
| Background/rationale | 2 | Explain the scientific background and rationale for the investigation being reported✅**P3** |
| Objectives | 3 | State specific objectives, including any prespecified hypotheses ✅**P3** |
| Methods | | |
| Study design | 4 | Present key elements of study design early in the paper ✅**P4** |
| Setting | 5 | Describe the setting, locations, and relevant dates, including periods of recruitment, exposure, follow-up, and data collection ✅**P4-5** |
| Participants | 6 | (*a*) Give the eligibility criteria, and the sources and methods of selection of participants ✅**P4** |
| Variables | 7 | Clearly define all outcomes, exposures, predictors, potential confounders, and effect modifiers. Give diagnostic criteria, if applicable ✅**P5-6** |
| Data sources/ measurement | 8* | For each variable of interest, give sources of data and details of methods of assessment (measurement). Describe comparability of assessment methods if there is more than one group ✅**P5-6** |
| Bias | 9 | Describe any efforts to address potential sources of bias ✅**P6** |
| Study size | 10 | Explain how the study size was arrived at ✅**P4** |
| Quantitative variables | 11 | Explain how quantitative variables were handled in the analyses. If applicable, describe which groupings were chosen and why ✅**P5-6** |
| Statistical methods | 12 | (*a*) Describe all statistical methods, including those used to control for confounding ✅**P6** |
|  |  | (*b*) Describe any methods used to examine subgroups and interactions ✅**P6** |
|  |  | (*c*) Explain how missing data were addressed ✅**P6** |
|  |  | (*d*) If applicable, describe analytical methods taking account of sampling strategy ✅**P6** |
|  |  | (*e*) Describe any sensitivity analyses |
| Results | | |
| Participants | 13* | (a) Report numbers of individuals at each stage of study—eg numbers potentially eligible, examined for eligibility, confirmed eligible, included in the study, completing follow-up, and analysed ✅**P7** |
|  |  | (b) Give reasons for non-participation at each stage ✅**P7** |
|  |  | (c) Consider use of a flow diagram ✅**P7** |
| Descriptive data | 14* | (a) Give characteristics of study participants (eg demographic, clinical, social) and information on exposures and potential confounders ✅**P7** |
|  |  | (b) Indicate number of participants with missing data for each variable of interest ✅**P7** |
| Outcome data | 15* | Report numbers of outcome events or summary measures ✅**P7** |
| Main results | 16 | (*a*) Give unadjusted estimates and, if applicable, confounder-adjusted estimates and their precision (eg, 95% confidence interval). Make clear which confounders were adjusted for and why they were included ✅**P8-14** |
|  |  | (*b*) Report category boundaries when continuous variables were categorized  ✅**P8-14** |
|  |  | (*c*) If relevant, consider translating estimates of relative risk into absolute risk for a meaningful time period |
| Other analyses | 17 | Report other analyses done—eg analyses of subgroups and interactions, and sensitivity analyses |
| Discussion | | |
| Key results | 18 | Summarise key results with reference to study objectives ✅**P14** |
| Limitations | 19 | Discuss limitations of the study, taking into account sources of potential bias or imprecision. Discuss both direction and magnitude of any potential bias ✅**P18-19** |
| Interpretation | 20 | Give a cautious overall interpretation of results considering objectives, limitations, multiplicity of analyses, results from similar studies, and other relevant evidence  ✅**P15-18** |
| Generalisability | 21 | Discuss the generalisability (external validity) of the study results ✅**P19** |
| Other information | | |
| Funding | 22 | Give the source of funding and the role of the funders for the present study and, if applicable, for the original study on which the present article is based ✅**P20** |
